# PReS-FINAL-2258: Final diagnoses of pediatric patients presenting with musculoskeletal symptoms in a center from the eastern Mediterranean

**DOI:** 10.1186/1546-0096-11-S2-P248

**Published:** 2013-12-05

**Authors:** O Cavkaytar, A Duzova, O Teksam, G Kale, S Ozen

**Affiliations:** 1 Department of Pediatrics, Hacettepe University Medical Faculty, Ankara, Turkey; 2 Department of Pediatric Nephrology and Rheumatology, Hacettepe University Medical Faculty, Ankara, Turkey

## Introduction

Complaints related with musculoskeletal system are frequent in children and adolescents.

## Objectives

To identify the clinical and laboratory features in children and adolescents suffering from musculoskeletal complaints (excluding acute traumatic conditions) in a tertiary referral center in Central Anatolia; and to define etiology and clues for differential diagnosis.

## Methods

All children [n: 422; mean age 7.90 ± 3.95 (range: 4 mo.- 18 years); 48.2% female] presenting to the outpatient clinic for the first time due to pain, swelling or limitation of movement attributed to musculoskeletal system in a 6 month period were enrolled. Demographical features, duration, and type of complaints, physical signs on initial presentation and laboratory findings [a complete blood count, erythrocyte sedimentation rate (ESR) and C-reactive protein (CRP)] were recorded.

## Results

Etiology was identified in 97.2% (n: 410) of the cases and were classified as follows: non-inflammatory and mechanical conditions (NIMC, n: 178; 42.2%), rheumatologic diseases (RD, n: 131; 31%), infection related disorders (IRD, n: 91; 21.6%) and malignancy (M, n: 10; 2.4%). NIMC group was characterized with longer duration of complaints, higher rate of non-articular complaints, lower rate of joint involvement, limping and lower levels of leukocytes, ESR, and CRP (p < 0.001). Rate of rheumatic disease was higher in >12 years of age group, compared to younger ones (p: 0.005). On the contrary younger age group was associated with higher rate of IRD group (p: 0.007). Small joint involvement was highest in RD group (16.8%), compared to other groups (p < 0.05). Rate of IRD was highest when the duration of complaints was less than 7 days, compared to the other groups (p < 0.05). Rheumatic disease had the lowest rate among patients with duration of complaints less than 7 days compared to the other three groups (p < 0.05). Familial Mediterranean fever (9.7%), juvenile idiopathic arthritis (8.3%) and Henoch-Schönlein purpura (5.7%) were the most frequent rheumatologic diseases. Median ESR levels in RD and M groups were higher, compared to IRD and NIMC groups (p < 0.05 respectively). Although mean ESR levels were comparable among M and RD groups, the frequency of patients with ESR levels ≥60 mm/hr were higher in M group (60%), compared to RD (20.6%; p <0.05) group.

## Conclusion

Rheumatologic diseases accounted approximately one third of the etiology among children and adolescents admitted with non-traumatic musculoskeletal complaints. Age, duration of complaints, pattern of joint involvement, and acute phase reactants are practical tools for the differential diagnosis.

## Disclosure of interest

None declared.

